# Antisuicidal (protective) factors in recovery from schizophrenia

**DOI:** 10.1192/j.eurpsy.2022.2174

**Published:** 2022-09-01

**Authors:** E. Lyubov, N. Semenova

**Affiliations:** 1 Moscow Research Institute of Psychiatry — Branch of The Serbsky National Medical Research Center for Psychiatry and Narcology, Suicidology Department, Moscow, Russian Federation; 2 Moscow Research Institute of Psychiatry, Clinical Psychology Counseling, Moscow, Russian Federation

**Keywords:** schizophrénia, Recovery, self-reports, antisuicidal factors

## Abstract

**Introduction:**

Determination of antisuicidal factors (AF) in balance with risk factors for suicidal behavior (SB) is essential for treatment and prophylactic measures.

**Objectives:**

Study AF in a sample of schizophrenic recovered patients (F.20, ICD-10) according to operational criteria R.P. Liberman et al. (2002).

**Methods:**

The content analysis of published self-reports of a sample (n = 13) of Russian and foreign psychiatrists and clinical psychologists with psychotic experience was used as a part of a more extensive qualitative analysis of «wounded healers».

**Results:**

In the history of > ½ (i.e., 7) ex-patients, repeated SPs (aborted suicides), as well as non-suicidal self-harm (e.g., self-cutting), were noted during the active period of the disease, and in four of them – during untreated psychosis. Following AFs can be distinguished in recovery state: clinical (absence of potentially suicidogenic residual depression or/and anxiety, according to criteria N.C. Andreasen et al. (2005) social (professional goals, coping with stigmatization), and existential (e.g., hope, gaining a whole Self ).

**Conclusions:**

AF is an important integral component of recovery in schizophrenia as a process of personality development despite a burden of severe mental disorders.

**Disclosure:**

No significant relationships.

